# Bronchoscopy Identifies Bacterial Airway Colonization and Comorbidities in Preschool Children With Refractory Respiratory Symptoms: A Retrospective Study

**DOI:** 10.1002/ppul.71435

**Published:** 2026-01-06

**Authors:** Helena Donath, Julius Ruff, Laura Heumüller, Olaf Eickmeier, Melanie Dressler, Ralf Schubert, Katharina Blumchen, Johannes Schulze, Stefan Zielen, Jordis Trischler

**Affiliations:** ^1^ Department of Pediatrics, Division of Pneumology, Allergology, Infectious Diseases and Gastroenterology Goethe University Frankfurt Frankfurt am Main Germany; ^2^ Respiratory Research Institute Medaimun GmbH Frankfurt am Main Germany

## Abstract

**Background:**

Preschool children with refractory respiratory symptoms often undergo diagnostic bronchoscopy to exclude anatomical and functional abnormalities and to detect suspected chronic lower respiratory tract infections by bronchoalveolar lavage (BAL). The objective of this retrospective analysis was to analyze BAL fluid findings with respect to bacterial colonization and cytology. Additionally, we aimed to determine associations with bacterial colonization of the airways and allergic sensitization status and symptoms as well as to identify comorbidities like gastroesophageal reflux disease (GERD) or eosinophilic esophagitis (EoE).

**Methods:**

In a retrospective analysis, the electronic medical records of 355 children aged 1–5 years who underwent bronchoscopy for refractory respiratory symptoms between 2010 and 2019 were evaluated. Differential cytology and bacterial cultures from BAL, laboratory parameters, oesophagogastroduodenoscopy (OGDS) and histology from esophageal biopsies were analyzed.

**Results:**

A positive bacterial culture from BAL fluid was found in 214 children (61.7%). Of these, 105 children (49%) subsequently received antibiotic treatment. The most frequently identified bacteria were *Haemophilus influenzae* (34%), *Streptococcus pneumoniae* (25%) and *Moraxella catarrhalis* (16%). The percentage of neutrophils in differential cell counts from BAL samples was significantly higher with positive bacterial cultures compared to negative cultures (29.2 + 28.1% vs. 21.2 + 25.4%, *p* = 0.02). Children with insufficient *S. pneumoniae* antibody titers had significantly more positive cultures for *S. pneumoniae* in BAL fluid (28.3% vs. 12.8%; *p* = 0.0024). GERD was identified in 115 children (32%) and EoE was diagnosed in nine children (2.8%).

**Conclusion:**

Bronchoscopy is a valuable diagnostic tool in the evaluation of persistent respiratory symptoms in preschool children. Bacterial colonization of the airways was common and associated with significantly elevated airway neutrophil counts.

**Abbreviations**
BAL: Bronchoalveolar lavage
CRP: C‐reactive protein
CT: Cycle threshold
ELISA: Enzyme‐linked Immunosorbent Assay
EoE: Eosinophilic esophagitis
GERD: Gastro eosophageal reflux disease
GINA: Global initative for asthma
H.: Haemophilus
ICS: Inhaled corticosteroids
IgE: Immunoglobulin E
IgG: Immunoglobulin G
IQR: Interquartile range
M.: Moraxella
PBB: Protacted bacterial bronchitis
PCR: polymerase chain reaction
PPI: Proton pump inhibitors
RSV: Respiratory syncytial virus
SD: Standard deviation
sIgE: Specific immunoglobulin E
SPT: Skin prick test
S.: Streptococcus

## 1 Introduction

Most preschool children with chronic or recurrent bronchitis are successfully treated for preschool wheeze with inhaled bronchodilators and inhaled corticosteroids (ICS) [1]. However, there is a subgroup of children who have recurrent or persistent, severe respiratory symptoms that are therapy refractory [2, 3]. These symptoms can range from obstructive episodes and chronic cough to recurrent pneumonias and protracted bacterial bronchitis (PBB), with a high rate of overlap [4]. According to current guidelines, these children are treated with courses of antibiotics and/or ICS [5, 6, 7]. If treatment is not successful and respiratory symptoms persist, these children often undergo bronchoscopy for diagnostic purposes. The aim is to improve treatment by detecting suspected chronic lower respiratory tract infections in bronchoalveolar lavage (BAL) fluid and to exclude rare causes of these refractory symptoms, like anatomical and functional abnormalities (e.g., trachea‐ and bronchomalacia, ‐stenosis). Additionally, by combining gastroscopy, alternate or aggravating causes for these symptoms, like gastroesophageal reflux disease (GERD) or eosinophilic esophagitis (EoE) can be assessed.

There is growing evidence that children who exhibit severe persistent preschool wheeze have increased bacterial colonization [8, 9] and altered colonization profiles in BAL fluid [10]. In a recent study, children with recurrent severe wheeze and allergic sensitization had a higher percentage of Moraxella (M.) catarrhalis detection [10]. Earlier retrospective studies in children with recurrent wheeze could detect *Haemophilus influenzae*, *M. catarrhalis* and *Streptococcus pneumoniae* [8, 9]. In the sequence of the etiopathogenesis, colonization occurs first, with bacteria being present and multiplying without causing symptoms. Infection can follow when bacterial growth, combined with impaired defenses, leads to inflammation and clinical illness [11]. However, it is unclear if bacterial colonization in wheezing preschool children leads to infection, is a mere indicator of immunological processes that increase susceptibility to wheezing, or maybe even predicts asthma diagnosis later in life [12].

Therefore, the objective of this retrospective analysis of diagnostic bronchoscopies in preschool children with persistent respiratory symptoms was to analyze BAL fluid findings regarding bacterial colonization and cytology. Moreover, we aimed to determine associations between bacterial colonization of the airways and allergic sensitization status, asthma severity, vaccination titers and to detect comorbidities like GERD and EoE.

## 2 Materials and Methods

### 2.1 Patients and Demographics

The present study was a retrospective cohort analysis of electronic patient records from a period of 10 years (01.01.2010‐31.12.2019) at the Department of Pediatrics, Division of Pneumology, Allergology, Infectious Diseases and Gastroenterology, University Hospital Frankfurt (Ethic commission reference number: 2022‐763). All patients aged 1–5 years who underwent inpatient bronchoscopy for chronic or refractory respiratory disease (recurrent or persistent wheeze, persistent cough, wet or productive cough, recurrent pneumonia with ≥ 3 episodes or > 1 month) were included. For subgroup analysis, patients were classified according to up to two main indications for bronchoscopy, based on the categories mentioned above. For the analysis, cases who underwent bronchoscopy due to foreign body aspiration, intensive care/intubation or known chronic pulmonary conditions such as cystic fibrosis, bronchopulmonary dysplasia, bronchiolitis obliterans, immunodeficiency, and so on were excluded. As a standard procedure at our center, children with chronic refractory disease also undergo gastroscopy for evaluation of GERD. We also did not include children younger than 1 year due to the higher prevalence of airway anomalies in this group. Cases were taken from the patient software ORBIS (Dedalus Healthcare GmbH, Bonn, Germany) when the procedural code for bronchoscopy was available. Additional clinical data were added via the program Medistar (CompuGroup Medical Deutschland AG, Koblenz, Germany).

Age, sex, length of stay, symptoms and medications (antibiotics, PPI and asthma medication) before and after bronchoscopy were taken from medical history. Asthma medication was grouped according to global initiative for asthma (GINA) guideline into steps 1–5 [7] and separated into groups for definition of asthma severity, with GINA steps 4 and 5 marking the most severe asthmatics.

### 2.2 Laboratory Parameters and Allergic Sensitization

The following laboratory parameters were recorded: c‐reactive protein (CRP), eosinophil granulocytes, immunoglobulin G (IgG) and its subclasses, pneumococcal antibody titers. Eosinophil granulocytes (blood) were considered pathological at values ≥ 300/µL. Vaccination protection against pneumococcus was defined as detection of titers > 1.0 μg/mL against at least three out of four serotypes (PNC6B, 7F, 19F, 23F) in enzyme‐linked immunosorbent assay (ELISA) [13].

Allergic sensitization was either documented as skin prick test (SPT) and/or specific immunoglobulin E (sIgE) against birch, grass, house dust mite, chicken egg protein, and cat. Sensitization was defined as positive reaction in SPT (defined by wheal size ≥ 3 mm) and/or detection of sIgE (defined as ≥ 0.35 kU/L).

### 2.3 Bronchoalveolar Lavage (Differential Cytology, Virology and Microbiology)

Differential cell counts on BAL were recorded and percentage of neutrophil granulocytes in BAL fluid ≥ 20% were defined as pathological, utilizing the upper range of normal in several healthy children cohorts [14, 15].

Bacterial detection was performed by culture‐based testing of BAL fluid with detection of any bacteria was considered positive (detection cut‐off ≥ 10³ CFU/mL).

Virus detection was done by polymerase chain reaction (PCR) with cycle threshold (CT) < 30 considered as positive.

### 2.4 Gastroscopy, pH Monitoring and Esophageal Biopsies

Gastroscopic findings were categorized into macroscopic esophagitis according to visual classification and microscopic esophagitis according to the pathological findings of biopsies from the esophagus.

Reflux in pH monitoring was divided into the following three categories: pH monitoring was considered pathological if the reflux index (percentage of time with pH < 4) was ≥ 5%. pH monitoring was considered as borderline with reflux index < 5%, but pathological DeMeester score or positive correlation with symptoms. pH monitoring was considered negative in case of reflux index < 5% and absence of symptoms [16].

GERD was defined as pathological pH monitoring or borderline pH monitoring and macroscopic and/or microscopic esophagitis.

Eosinophilic esophagitis was defined as more 15 Eosinophils/high power field (HPF) as by international guidelines [17].

### 2.5 Statistical Analysis

For this retrospective study GraphPad Prism Version 10 (GraphPad Software Inc., La Jolla, CA, USA) was used for statistical analyses. Data is presented as mean and standard deviation (SD) or as median and range respectively, according to the Kolmogorov‐Smirnov test for normal distribution. Inter‐group comparisons were calculated by unpaired *t*‐test or Mann‐Whitney test. *p* < 0.05 was considered as statistically significant. Statistical analysis on categorial values were only performed on ordinal data that were treated as ranks (ordinal data) and therefore Mann‐Whitney test was performed. To assess associations between categorical variables, a Chi‐square test of independence was applied to the contingency table data.

## 3 Results

### 3.1 Patient Characteristics

**FIGURE 1:**
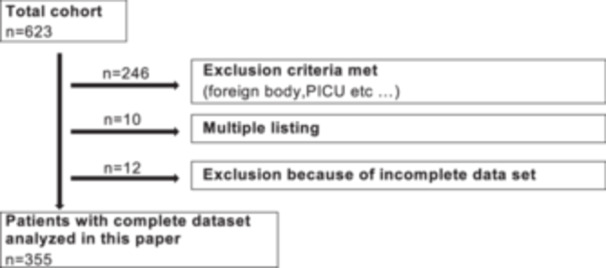
Flowchart of patient selection.

A total of 355 patients were included in this retrospective study. The flowchart regarding the patient selection is shown in Figure 1.

213 (60%) were boys and 142 (40%) were girls. The median age at admission was 32 months (IQR 22–49). The median length of stay was 3 days (IQR 2–5). Two hundred two patients (56,9%) had only one symptom category as main indication, recurrent and persistent wheeze being the most prevalent (62 patients, 17,5%). The rest had an overlap of symptom categories, recurrent and persistent wheeze plus another symptom was the most common (105 patients, 29,5%), see Table 2.

Blood eosinophil count was obtained in 343 patients (96.6%). A pathological value ≥ 300/µL was present in 128/343 (37.3%) of patients. Allergy testing was performed in 254 patients (71.5%) by sIgE and 311 patients (87.6%) by SPT. Sensitization was detected in 78 patients (22%) by SPT or sIgE. Total IgE was significantly higher in the group with high sensitization than in the group without sensitization (84 U/mL (IQR 45.5–169.25 U/mL) vs. 21 U/mL (IQR 7–55 U/mL), *p* < 0.0001, Mann‐Whitney U).

Pneumococcal antibodies were determined in 254 patients (71.5%), of whom 141 (55.5%) had sufficient vaccination protection. Overview of patient characteristics is shown in Table 1.

**TABLE 1:** Patient characterization.

Total cohort (n)	355
Sex (Male/Female), (% of total cohort)	213/142 (60/40)
Age in months (IQR)	32 (22–49)
Length of stay in days (IQR)	3 (2–5)
Total IgE, number tested (% of total cohort)	319 (89.9)
– Median (U/mL)	33 (IQR 10–82.5)
IgG, number tested (% of total cohort)	319 (89.9)
– Median (mg/mL)	724 (IQR 616.5–832.5)
CRP, number tested (% of total cohort)	354 (99.7)
– Median (mg/dL)	0.05 (IQR 0.02–0.12)
Leukocytes, number tested (% of total cohort)	353 (99.4)
– Median (/nL)	8.66 (IQR 7.23–10.67)
Eosinophiles, number tested (% of total cohort)	350 (98.6)
– Median (/nL)	0.22 (IQR 0.14–0.4)
Gastroscopies (% of total cohort)	341 (96.1)
Asthma medication before (% of total cohort)	300 (84.5)
Diagnosis of atopic dermatitis (% of total cohort)	30 (8.5)
Detection of sensitization via SPT or sIgE^a^, number tested (% of total cohort)	342 (96.3)
– Positive sensitization (% of total cohort)	78 (22)
Pneumococcal antibodies, number tested (% of total cohort)	254 (71.5)
– Sufficient vaccination protection (% of total cohort)	141 (39.7)

*Note:* Values are presented as median with IQR (Q1–Q3).

^a^ Allergens tested: mite, birch, grass, milk, egg, cat hair, horse hair.

**TABLE 2:** Main indications for bronchoscopy (symptom categories).

Main indication for bronchoscopy	Patient n (%)	Positive cultures n (%)	GERD (%)
Any indication	355 (100%)	214 (61,7%)	115 (32,4%)
Any wet cough/PBB – With wheeze – With pneumonia – With cough	112 (31,5%) – 33 – 26 – 5	72 (64,9%)	43 (38,4%)
Only wet cough/PBB	48 (13,5%)	33 (70,2%)	20 (41,7%)
Any recurrent wheeze – With wet cough – pneumonia – With cough	167 (47%) – 33 – 48 – 24	103 (64,4%)	57 (34,1%)
Only recurrent wheeze	62 (17,5%)	37 (62,7%)	21 (33,9%)
Any recurrent pneumonia – With wet cough – With wheeze – With cough	122 (34,4%) – 26 – 48 – 17	65 (55,1%)	32 (26,2%)
Only recurrent pneumonia	31 (8,7%)	11 (35,5%)	6 (19,4%)
Any cough – With wet cough – With wheeze – With pneumonia	107 (30,1%) – 5 – 24 – 17	68 (63,6%)	33 (30,8%)
Only cough	61 (17,2%)	37 (60,7%)	18 (29,5%)

### 3.2 Microbiological and Virological BAL Findings

**FIGURE 2:**
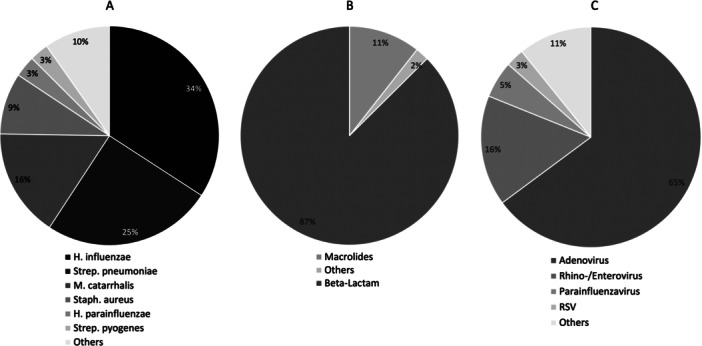
(A) Percentages of all detected bacteria; (B) Percentage of prescripted antibiotics after bronchoscopy; (C) Percentages of all detected viruses.

Three hundred forty‐seven cultures of BAL fluid were performed. Of these, a bacterium was detected in 214 (61.7%). The distribution of the detected bacteria is shown in Figure 2A. H. influenzae was the most common bacterium in 34% (*n* = 98) of children, followed by *S. pneumoniae* (25%, *n* = 70) and *M. catarrhalis* (16%, *n* = 46).

Eosinophils were not significantly elevated in patients, who had positive cultures for M. catarrhalis (240/µL (IQR 150–340/µL) vs. 240/µL (IQR 140–430/µL), *p* = 0.43). Of all positive cultures for M. catarrhalis, it was detected in 37/46 (80.4%) of children receiving asthma therapy at GINA steps 4 and 5.

Patients with only recurrent pneumonia as main indication had significantly less positive BAL cultures compared to the overall population (35.5% vs. 61.7%, *p* < 0,05).

Among patients with sufficiently high antibodies against *S. pneumoniae*, *S. pneumoniae* grew in 18 (12.8%) of the BAL fluid‐culture based sterility tests whereas S. pneumoniae was detected in 32 patients without vaccination protection (28.3%) (*p* = 0.0024, Mann‐Whitney U). No protection against pneumococcal antigen was present in 31.8% (*n* = 113) of the cohort.

A total of 215/355 samples (60.5%) were examined by virus PCR. 181 (84.2%) were negative, in 34 (18.8%) a virus was detected. The distribution is shown in Figure 2C. The most frequently detected virus was adenovirus with 65% (*n* = 24).

### 3.3 BAL Cytology

**FIGURE 3:**
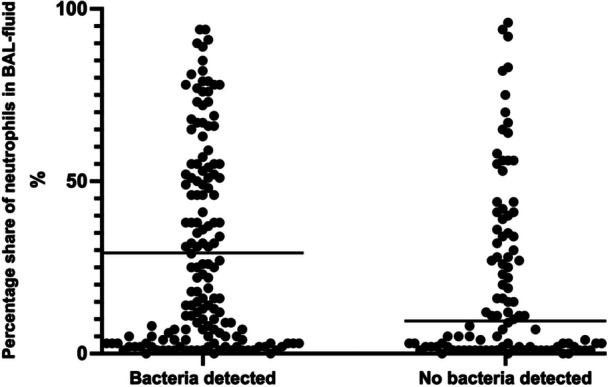
Neutrophils in patients with and without bacterial detection; *p* = 0.0225; Mann‐Whitney U test.

Airway neutrophil count was available from 248/347 patients (71.5%). 151 patients (60.9%) with bacterial detection and 97 patients (39.1%) without bacterial detection on BAL had neutrophil counts. There was a significant difference between airway neutrophil counts in bacteria positive versus bacteria negative BAL fluid (Figure 3), (18% (IQR 3.5%–51%) vs. 9% (IQR 2%–34%), *p* = 0.023). No difference between peripheral blood eosinophil and leukocyte counts in patients with neutrophilic airway inflammation was observed.

### 3.4 Antibiotic Treatment

A total of 146 (41.1%) patients received antibiotic treatment after bronchoscopy. In 71.9% of antibiotic‐treated patients (*n* = 105), bacteria were detected in the BAL fluid. The most common antibiotics prescribed after bronchoscopy were beta‐lactam antibiotics (87%).

Before bronchoscopy, 124 (34.9%) patients were treated with antibiotics. The most prescribed antibiotic was azithromycin in 101 (81.5%) cases. Bacteria were still detected in the BAL fluid of *n* = 71/124 (57.3%) pretreated patients.

Figure 2B shows the distribution of antibiotic therapy after bronchoscopy.

### 3.5 Asthma Therapy

A total of 300 patients (84.5%) received asthma therapy before bronchoscopy (GINA step 1: *n* = 11, step 2 + 3 *n* = 75, step 4 + 5 *n* = 214). After bronchoscopy, 328 (92.4%) received asthma therapy (GINA step 1: *n* = 2, step 2 + 3 *n* = 70, step 4 + 5 *n* = 256).

Two hundred fifty‐six patients had asthma therapy step 4 or more according to GINA guidelines. Of these patients, 37 (14%) had *M. catarrhalis* and 6 (2.3%) rhinovirus in BAL fluid.

### 3.6 GERD and PPI Therapy

Three hundred forty‐one patients (96.1%) underwent gastroscopy. In 229/355 of patients (64.5%) pH monitorings were performed. Sixty‐nine (30.1%) exhibited pathological findings, 57 (24.9%) were borderline pathological, and 103 (45%) had negative results. Patients with pathological pH monitoring exhibited an abnormal macroscopic finding on gastroscopy in 59.4% and had confirmatory positive histology obtained during gastroscopy in 52.2%. PPI therapy was given to 91.3%, 70.2%, and 48.6% of patients with pathological, borderline, and normal pH monitoring, respectively.

### 3.7 EoE

In total 324 patients had a biopsy performed during their gastroscopy. Of these, 9 (2.8%) patients suffered from histologically confirmed eosinophilic esophagitis (EoE). Prior to endoscopy, all EoE patients had asthma therapy (*n* = 2 with GINA stage 2 + 3, *n* = 7 with GINA stage 4 + 5). PH monitoring was pathological in 5/9 EoE patients. After endoscopy, 7/9 patients (77.8%) were treated with PPI, none was pre‐treated with PPI. Allergic sensitization was present in three EoE patients. Laboratory chemistry showed a median peripheral eosinophil count at 0.93/nL (IQR 0.59–1.15/nL) and an increased IgE of 55 kU/L (IQR: 30–83 kU/L).

## 4 Discussion

In the present study, bacterial colonization of the airways was detected in approximately 60% of BAL fluids. This finding is relevant, as the BAL fluids were not collected from children with acute pulmonary infections but from preschool children with recurrent respiratory symptoms like recurrent or persistent wheeze, wet cough, recurrent pneumonia or chronic cough. The growing recognition of airway colonization in the pathogenesis of asthma is supported by several studies demonstrating an association between lower airway colonization with pathogens such as *M. catarrhalis*, *H. influenzae*, or *S. pneumoniae* in children and asthma symptoms [8, 9]. Interestingly, our study showed no difference in percentage of positive cultures and pathogen variety between different symptom category groups, except patients who underwent the procedure only because of recurrent pneumonias. This is supported by similar studies looking at smaller cohorts of preschool children with overlapping respiratory symptoms [4, 18]. Therefore, this large cohort of preschool children presented here adds to existing data and challenges treating physicians to rethink antibiotic treatment in these cases, even if a persistent wheeze is the main symptom.

Only few studies of bacterial cultures from BAL of healthy children have been published, with varying results, generally showing less bacterial growth and different species [8, 19]. Due to ethical reasons, BAL in completely healthy children is rare, and children often undergo bronchoscopy for different indications. The authors chose to define any positive bacterial culture (> 10³ CFU/mL) as bacterial colonization of the lower airways. The definition of a positive BAL culture yield is heterogenous, varying from 10³ CFU/mL to 10^5^ CFU/mL [20]. It must be emphasized that a detection threshold of > 10^5^ CFU/mL or > 10^4^ CFU/mL is widely considered a bacterial infection [21, 22]. It is therefore neither possible nor the intention of this study to discriminate between bacterial infection and colonization. PCR for the detection of various viral and bacterial pathogens were not available for the complete dataset.

A recent study by Robinson et al. has proposed a characterization of wheeze endotypes in a prospective workup of bronchoscopies [10]. This group has singled out *M. catarrhalis* as a risk factor for severe courses in preschool wheeze. This hypothesis is supported by researchers who have demonstrated a possible co‐infection with *M. catarrhalis* and *H. influenzae* during severe respiratory syncytial virus (RSV) infection in the first 6 months of life as a risk factor for subsequent preschool asthma [23]. In the present cohort of preschool children, the majority had asthma therapy. However, there was no specific endotype as defined by blood eosinophilia that was associated with an obvious pattern of colonization. Yet, M. catarrhalis detection was more likely in preschool wheeze with high asthma therapy intensity (GINA step 4 and 5), indicating more severe asthma.

We demonstrated that *S. pneumoniae* was detected significantly more often in the lower airways of children who had low pneumococcal vaccination titers, meaning they were either not vaccinated or did not respond adequately. Increased pneumococcal carriage in the nasopharynx of asthmatic children has been demonstrated before, but a correlation with vaccination status has not been shown so far [24]. This supports the protective effect of a vaccination also against colonization, and the presented results may indicate that risk groups might potentially benefit from an additional booster vaccine. This has been suggested in a recent study, which demonstrated that a booster vaccine was able to reduce exacerbations in asthmatic preschool children who did not have sufficient vaccination titers [25].

Similar to previous studies, we also demonstrated that positive bacterial BAL fluid cultures are associated with airway neutrophilia [8, 9]. Neutrophilic inflammation is a risk factor for the development of bronchiectasis [26]. In addition, airway neutrophilia is found more frequently in patients with refractory asthma [8, 9].

Before bronchoscopy, 84.5% of children were already receiving asthma therapy. After bronchoscopy, this proportion increased to 92.4%. In particular, the proportion of patients receiving therapy according to GINA Guidelines steps 4 and 5 increased. This increase is most likely due to the patients who did not have alternating or aggravating findings on bronchoscopy and gastroscopy that would lead to alternative therapies.

In the current study, 34.9% of children with chronic symptoms received antibiotic therapy before bronchoscopy. However, despite antibiotic pre‐therapy, a bacterium was detected in 57.3% of pretreated patients. The most frequently prescribed antibiotic was azithromycin. Although this is not in accordance with most guidelines for PBB [27], it is still common practice in pediatric pulmonology despite ongoing efforts by antibiotic stewardship programs. Apart from its microbial effect, azithromycin also has anti‐inflammatory properties, and few studies showed a positive effect on infection‐triggered wheeze in preschoolers [28]. Nevertheless, this must be critically evaluated considering the increased incidence of macrolide resistance in common respiratory pathogens. Moreover, considering the presented microbiological findings, azithromycin does not seem to be the right antimicrobial agent and antibiotic treatment for 2–4 weeks according to the international PBB guidelines should therefore be initiated when appropriate [29].

GERD is known to have a higher prevalence in children with persistent asthma [30]. Overall, though, the incidence of GERD seems to decrease during infancy [31]. Typical symptoms can be absent, and the diagnosis is therefore challenging. The current diagnostic recommendation is esophageal multichannel intraluminal impedance testing [32]. Nevertheless, pH monitoring, as well as histological examination of biopsies are still a diagnostic pillar [32]. In infancy there is little to no evidence about when and how to treat [33]. The only consensus is that PPI should be used critically in this vulnerable age group, as side effects are considerable including frequent pulmonary infections due to lowered acid barrier. Recently, Teague et al. were able to identify a cluster of patients with preschool wheeze that has a high percentage of GERD and lipid‐laden macrophages due to microaspirations [34]. Whereas in this study GERD was only described by symptoms, we were able to define the diagnosis by pH monitoring and gastroscopy including histological samples. Combined, this strengthens the need to evaluate GERD in cases of refractory pulmonary symptoms.

Finally, the high rate of EoE detections should be emphasized. The stated incidence in Europe and North America of 6.6/100,000 children [35] was exceeded in the present investigation with 2.5% of the total cohort. A combined gastroscopy and bronchoscopy appears to be reasonable in refractory respiratory symptoms despite multimodal therapy and with indications of reflux, especially in males with peripheral eosinophilia and elevated IgE, particularly since symptoms of EoE in preschool children can be diverse and include chronic cough.

The current study has limitations, primarily due to its retrospective design, resulting in a heterogeneous cohort and lack of long‐term follow up. However, this heterogeneity is also partly inherent to these diseases, this is illustrated by the similiarity of outcomes of the different symptom categories in this study. Moreover, studies examining asthma and PBB separately have found an increased likelihood of having antibiotics prescribed for PBB or pneumonia in asthmatics [36] and conversely, children with PBB are about twice as likely to suffer from asthma than healthy children [6].

## 5 Conclusion

Bronchoscopy is a valuable diagnostic tool in persistent respiratory symptoms in preschool children regardless of symptom categories. Airway bacterial colonization was common across the cohort and was associated with significantly elevated airway neutrophil counts. In a substantial number of cases, bronchoscopic findings led to changes in clinical management, like targeted antibiotic treatment, initiation of antireflux therapy and increased asthma medication. These therapy adjustments should be implemented consistently and further prospective studies that analyze the outcome of implementing treatment changes after bronchoscopy are needed. In addition, assessing pneumococcal titers is reasonable in cases of persistent respiratory symptoms in children, and combined gastroscopy and pH monitoring can find comorbidities like GERD and EoE more often in this cohort than in the general population.

## Author Contributions

**Helena Donath:** writing – original draft, formal analysis. **Julius Ruff:** data curation, visualization, writing – review and editing. **Laura Heumüller:** data curation. **Olaf Eickmeier:** investigation, data curation. **Melanie Dressler:** investigation, data curation. **Ralf Schubert:** investigation. **Katharina Blumchen:** writing – review and editing, investigation, supervision. **Johannes Schulze:** investigation, data curation. **Stefan Zielen:** conceptualization, writing – review and editing, supervision, resources, methodology. **Jordis Trischler:** conceptualization, writing – original draft, validation, formal analysis, supervision, methodology, writing – review and editing.

## Funding

The authors received no specific funding for this work.

## Conflicts of Interest

The authors declare no conflicts of interest.

## Data Availability

The data that support the findings of this study are available from the corresponding author upon reasonable request.
